# More Prominent Nonlinear Mixed Selectivity in the Dorsolateral Prefrontal than Posterior Parietal Cortex

**DOI:** 10.1523/ENEURO.0517-21.2022

**Published:** 2022-04-25

**Authors:** Wenhao Dang, Sihai Li, Shusen Pu, Xue-Lian Qi, Christos Constantinidis

**Affiliations:** 1 Department of Biomedical Engineering, Vanderbilt University, Nashville, TN 37235; 2 Department of Neurobiology, University of Chicago, Chicago, IL 60637; 3 Department of Neurobiology and Anatomy, Wake Forest School of Medicine, Winston-Salem, NC 27157; 4 Neuroscience Program, Vanderbilt University, Nashville, TN 37235; 5 Department of Ophthalmology and Visual Sciences, Vanderbilt University Medical Center, Nashville, TN 37232

**Keywords:** mixed selectivity, parietal, prefrontal, working memory

## Abstract

Neurons in the dorsolateral prefrontal cortex (dlPFC) and posterior parietal cortex (PPC) are activated by different cognitive tasks and respond differently to the same stimuli depending on task. The conjunctive representations of multiple tasks in nonlinear fashion in single neuron activity, is known as nonlinear mixed selectivity (NMS). Here, we compared NMS in a working memory task in areas 8a and 46 of the dlPFC and 7a and lateral intraparietal cortex (LIP) of the PPC in macaque monkeys. NMS neurons were more frequent in dlPFC than in PPC and this was attributed to more cells gaining selectivity in the course of a trial. Additionally, in our task, the subjects’ behavioral performance improved within a behavioral session as they learned the session-specific statistics of the task. The magnitude of NMS in the dlPFC also increased as a function of time within a single session. On the other hand, we observed minimal rotation of population responses and no appreciable differences in NMS between correct and error trials in either area. Our results provide direct evidence demonstrating a specialization in NMS between dlPFC and PPC and reveal mechanisms of neural selectivity in areas recruited in working memory tasks.

## Significance Statement

How the activity of neurons mediates performance of multiple cognitive tasks and where this is implemented in the brain remain unclear. One popular theory suggests that nonlinear mixed selectivity (NMS), altered tuning for the same stimuli depending on task or rule, allows neurons to represent multiple tasks contexts. We wished to test whether NMS differed systematically between two areas that have been implicated in cognitive tasks, the dorsolateral prefrontal cortex (dlPFC) and posterior parietal cortex (PPC). Our results reveal only limited nonlinear selectivity in the two areas, though more prominent in the dlPFC. Our results constrain theories of the neural basis of cognitive function.

## Introduction

Neurons at the highest levels of the cortical hierarchy need to represent not only sensory stimuli but also the significance each stimulus has in the context of the current task, to achieve the level of adaptability that is necessary for complex behavior (Riley and Constantinidis, 2016). It has been proposed that this neuronal property depends on nonlinear mixed selectivity (NMS), the combination of responses to multiple internal and external variables in a nonlinear fashion (Rigotti et al., 2013). The prefrontal cortex (PFC) in particular has been implicated in increased cognitive flexibility as a result of training (Constantinidis and Klingberg, 2016) and of cognitive maturation in adolescence (Constantinidis and Luna, 2019). Individual neurons in the PFC typically encode multiple stimulus dimensions and task rules during performance of a task (Asaad et al., 2000; Mansouri et al., 2006; Machens et al., 2010; Warden and Miller, 2010; Qi et al., 2015). A proportion of prefrontal neurons indeed exhibit NMS for these variables (Rigotti et al., 2013; Parthasarathy et al., 2017; Johnston et al., 2020; Dang et al., 2021). These findings however have not been universal. Some studies have failed to detect appreciable numbers of neurons with NMS (Cavanagh et al., 2018; Dang et al., 2021). It is an open question therefore whether NMS is essential to perform any complex task.

The PFC is only part of a broader network activated during cognitive functions such as working memory (Constantinidis and Procyk, 2004). The posterior parietal cortex (PPC), in particular, has long been known to be active in many of the same tasks, including working memory (Gnadt and Andersen, 1988; Constantinidis and Steinmetz, 1996; Chafee and Goldman-Rakic, 1998; Li et al., 2021). This is not to say, however, that response patterns in the two areas are identical. PFC exhibits unique properties including differential ability to represent the location of a remembered stimulus when distracting stimuli are present (di Pellegrino and Wise, 1993; Constantinidis and Steinmetz, 1996; Qi et al., 2010; Suzuki and Gottlieb, 2013), although the specific patterns of responses in the two areas appear to be task-dependent (Jacob and Nieder, 2014; Qi et al., 2015). These differences in functional properties, in turn, can be directly attributed to differences in intrinsic circuits in the two areas (Zhou et al., 2012; Katsuki et al., 2014; Hart and Huk, 2020).

We were thus motivated to examine whether NMS differs systematically between the PFC and PPC and whether potentially higher levels of prefrontal NMS can account for greater capacity for plasticity. Some evidence exists for higher nonlinear integration of remembered stimuli with newly appeared ones in the PFC (Zhou et al., 2021); however, it is unknown whether the finding is task specific. Therefore, we analyzed data from monkeys performing working memory tasks involving comparison of two stimuli presented in sequence and thus requiring a flexible representation of a stimulus depending on whether it appeared first in the sequence (cue) or second (match). Our task also allowed monkeys to infer the statistics of stimulus location during the course of a daily behavioral session, providing a second opportunity to determine how this flexibility is implemented at the neuronal level and whether its time course differs between areas.

## Materials and Methods

### Test subjects

Behavioral and neurophysiological results were obtained from two male rhesus monkeys (*Macaca mulatta*) weighing 9–12 kg. All experimental procedures followed guidelines set by the United States Public Health Service *Policy on Humane Care and Use of Laboratory Animals*, and the National Research Council’s *Guide for the Care and Use of Laboratory Animals* and were reviewed and approved by the Wake Forest University Institutional Animal Care and Use Committee. Additionally, this study was conducted in compliance with the ARRIVE guidelines.

### Experimental setup

Monkeys sat in a primate chair with their head fixed while viewing an LCD monitor positioned 68 cm away from their eyes in dim ambient illumination. Animals were required to fixate on a 0.2° white square that appeared in the center of the monitor screen through each trial while visual stimuli were presented. Breaking fixation would result in immediately terminating the trial without reward. Eye position was monitored throughout the trial using a noninvasive, infrared eye position scanning system (model RK-716; ISCAN). The presentation of visual stimuli, the monitoring of eye position, and the synchronization of stimuli with neurophysiological data were performed with in-house software (Meyer and Constantinidis, 2005) implemented in the MATLAB environment (MathWorks).

Two 20-mm diameter craniotomies were performed over the right lateral PFC and PPC, over which recordings were possible in areas 8a and 46 of the posterior-dorsal and mid-dorsal subdivisions of the PFC (Riley et al., 2017), and areas 7a and LIP of the PPC, respectively. Neurophysiological recordings were obtained with tungsten-coated electrodes with a 200 or 250 μm in diameter and 4-MΩ impedance at 1 kHz (FHC). Arrays of up to 4-microelectrodes spaced within 1 mm of each other were advanced into the cortex with a Microdrive system (EPS drive, Alpha-Omega Engineering) through the dura into the cortex (Zhou et al., 2016). The signal from each electrode was amplified and bandpass filtered between 500 Hz and 8 kHz while being recorded with a modular data acquisition system (APM system, FHC). Waveforms that exceeded a user-defined threshold were sampled at 25-μs resolution, digitized, and stored for off-line analysis.

### Behavioral task and stimuli display

Monkeys were trained to perform the Oculomotor Delayed Response (ODR) task and the Match-Stay Nonmatch-Go (MSNG) task. In both tasks, a trial started with a fixation point at the center of the screen. After gaze was maintained on the fixation point for 1.0 s, a cue stimulus appeared for 500 ms. The stimulus consisted of a 1° white square, which appeared at a pseudorandom location, at an eccentricity of 10°. This was followed by a delay period. In the ODR task, the delay period lasted for 1.5 or 3 s, after which the animals were required to make a saccade to the remembered location of the cue, which could appear at one of eight locations. In the MSNG task, the delay period lasted for 3 s, after which a second stimulus appeared either at the same location as the cue, and constituted a match, or at a different, reference location and constituted a nonmatch. The fixation point changed color 500 ms after the appearance of the second stimulus and the monkey was required to maintain fixation if it was a match or to make a saccade toward the stimulus if it was a nonmatch. Possible cue locations included a reference location, which differed from session to session, and eight locations deviating from the reference location by an angular distance of 11.25°, 22.5°, 45°, and 90°, either clockwise or counterclockwise. The match would appear at the same location as the cue in approximately half the trials (9/17 conditions). The nonmatch would appear only at the reference location in the rest of the trials (8/17 conditions). Monkeys were rewarded with a liquid reward in both tasks. Based on the estimated best neuronal responding location in the ODR task, we selected the locations of stimuli used in the MSNG task, aiming to place the reference location at the flank of an isolated neuron’s receptive field. However, recordings were obtained from neurons at multiple electrodes, and the stimulus location could appear anywhere relative to a neuron’s receptive field.

### Data analysis

All analyses in the current study used behavior and neural data from the MSNG task and was performed in the MATLAB environment (MathWorks). We expressed performance in the MSNG tasks as the percentage of completed trials that resulted in correct responses. Some trials were aborted early because of breaks in fixation, blinks, or premature saccades that occurred before the fixation point changed color; these were ignored in performance estimation. To quantify the behavior improvement in a single experimental session, the percentage correct was calculated in nonoverlapping pseudo-blocks with 40 finished trials. Results from behavioral sessions with at least 160 finished trials were included in this analysis.

Recorded spike waveforms were sorted into separate units using a semi-automated cluster analysis process relying on the KlustaKwik algorithm (Harris et al., 2000). To classify neurons of the MSNG task into different categories of selectivity, we performed ANOVA tests to investigate how neurons encode various combinations of different task variables. We first performed a two-way ANOVA to determine the influence of task epoch (first vs second stimulus presentation epoch) on the neuron’s spatial turning (Dang et al., 2021). The two ANOVA factors were the visual stimulus location and the task epoch (cue or match period) in which the stimulus appeared. The dependent variable was the neuron’s average firing rates across stimulus presentation epochs. Neurons with classic selectivity (CS) exhibited a main effect of only one factor without any significant interaction term, evaluated at the α = 0.05 significance level. Neurons with linear mixed selectivity (LMS) exhibited main effects of both factors without a significant interaction term. Neurons with NMS exhibited a significant interactions term. Finally, nonselective (NS) neurons exhibited no significant main effect or interaction term. Cells with at least six match trials for each location were included in this analysis. Similarly, we also performed a two-way ANOVA to determine the influence of spatial tuning on the neuron’s reward response. The two ANOVA factors were the visual stimulus location in the cue period and if the trial was rewarded or not. The dependent variable was the neuron’s average firing rate in the 500-ms window after the reward time for the trial. For this analysis, we chose correct trials from the match condition and error trials from the nonmatch condition for comparison, to avoid confounds caused by saccades right before the reward. To be included in this analysis, a neuron must have had at least four correct and error trials in at least three cue locations. To quantify location selectivity in the cue and the delay period, an one-way ANOVA tests were used to quantify location selectivity in the cue and the delay period. The mean firing rates in the cue and the delay period from all trials were used to fit a Gaussian function to estimate the tuning curve of the cell. The center of the Gaussian was defined as the preferred location of the cell.

We used a permutation test to evaluate differences in CS across the dlPFC and PPC. First, we calculated the proportion of both types of CS cells (selective for location/task epoch) in both areas. Then, in each iteration, we resampled the original dlPFC and PPC cell pool, controlling the proportion of CS cells to be the same as the calculated ratio in either area. A total of 500 iterations were conducted for each area and for each CS proportion combination.

We repeated the ANOVA analysis on subsets of data obtained at different time points during the course of each behavioral session, to quantify changes in the proportion of NMS cells. A three-trial long sliding window was then used to construct a subset of trials (three trials for nine locations, thus 27 trials for each time point) for the ANOVA from each stimulus location in chronological order. A nonparametric permutation test was applied to examine the statistical significance of changes in NMS during each session. The trial order was shuffled for every stimulus location in each iteration of the permutation test. The same sliding window sampling procedure and ANOVA test was then performed for each iteration. The F-score for the interaction term of the ANOVA analysis was linearly regressed to trial order. The distribution of regression slopes from the randomly shuffled sample was compared with that of the original data. The difference was deemed to be significant if the empirically observed slope occurred at the extremes of this null distribution (*p* < 0.05, two-tailed).

To further analyze the composition of NMS cells across the dlPFC and PPC, we categorized NMS based on their selectivity in the cue and match period. For this analysis, we ran a one-way ANOVA comparing firing rates of single NMS cells to different locations in the cue and match periods and calculated cue-selective, match-selective, and dual-selective NMS cells (*p* < 0.05 for each comparison).

We used the *F* statistic of the ANOVA interaction term to compare NMS in correct and error trials. This analysis exclusively used neurons with at least four trials in both the correct and error datasets in at least three different stimulus locations. The number of minimum trials and stimulus locations was chosen to maximize the average trial numbers for each selected cell while retaining a sufficiently large sample (>100 cells). The same number of trials from each stimulus location were randomly chosen in the correct and error dataset. This randomized trial selection process was repeated 50 times to minimize the variability because of the uneven number of available trials in the two datasets.

We applied principal components analysis (PCA) to visualize the neural population activity manifolds during the MSNG tasks. PCA was performed on the mean firing rate in the cue and the match period for the match trials. Only neurons with >8 trials at each location were included in the analysis. We organized the mean firing rates in the match trials of MSNG task into a 16×N matrix, where 16 is the number of conditions (eight locations × cue/match) and N is the number of neurons. We then performed dimensionality reduction using singular value decomposition (SVD), implemented in MATLAB, which yielded the principal components of the population response. We selected the first three eigenvectors for visualization. In the 3D PCA space, the representations of the eight locations in both the cue and the match period roughly formed a two-dimensional plane (as shown in Fig. 4). The angle of two planes $P_{1}$ and $P_{2}$ was then calculated using the following equation:

$$P_{1},P_{2}=\cos^{-1}\left(\frac{\left|({\vec{v}}_{1}\times {\vec{v}}_{2})\cdot ({\vec{v}}_{3}\times {\vec{v}}_{4})\right|}{\left|{\vec{v}}_{1}\times {\vec{v}}_{2}|\cdot |{\vec{v}}_{3}\times {\vec{v}}_{4}\right|}\right),$$

where “ $〈P_{1},P_{2}〉$” denotes the angle between $P_{1}$ and $P_{2}$, and “ ${\vec{v}}_{1}\times {\vec{v}}_{2}$” is the cross product that finds the vector perpendicular to the plane spanned by ${\vec{v}}_{1}$ and ${\vec{v}}_{2}$. To estimate the variance of the rotation angles, we resampled our PFC and PPC dataset 100 times, each time randomly picking half of the cells in the dataset to calculate the rotation.

## Results

**Figure 1.**
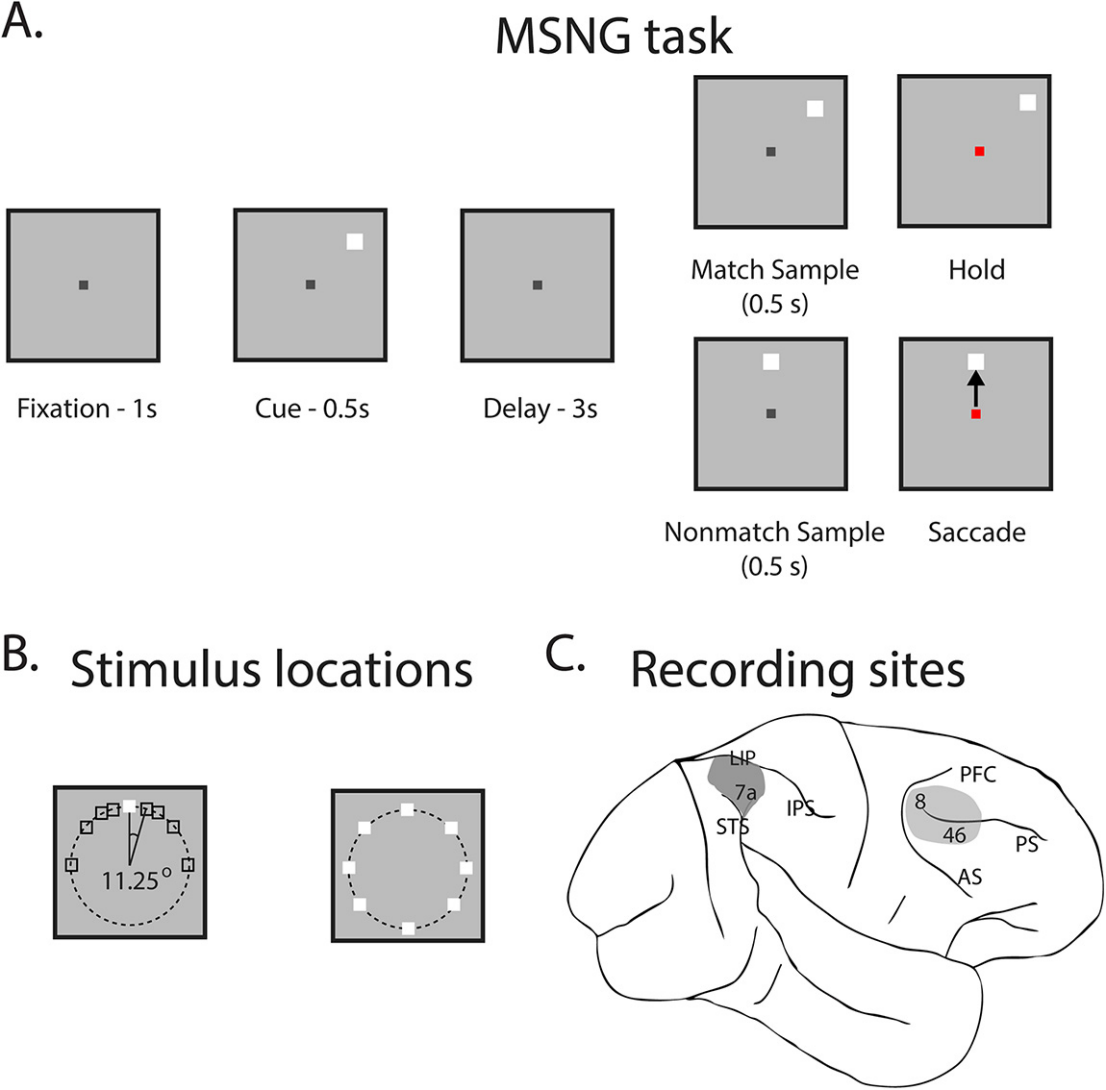
Tasks and areas for neurophysiological recordings. ***A***, Frames represent the sequence of event during the MSNG task, in which they were required to report whether two visual stimuli appeared at a matching spatial location, by either continuing to fixate on the center fixation point (match trials), or by saccading to the location (nonmatch trials) of the second stimulus. ***B***, The possible locations of stimuli were arranged in an invisible half circle with 10° of visual angle eccentricity, and the presentations of the stimuli were separated by a 3-s delay period in which no stimuli were displayed. The first stimulus could appear pseudo-randomly at one of nine possible spatial locations. Possible cue locations included a reference location (shown here at the top location) and eight locations deviating from the reference location by an angular distance of 11.25°, 22.5°, 45°, and 90°, either clockwise or counterclockwise. The reference location was changed from session to session and could appear in eight locations 45° apart around the fixation point (see Materials and Methods). This cue was followed by a second stimulus (henceforth referred to as the match or nonmatch). The match would appear at the same location as the cue in approximately half the trials (9/17 conditions). Similarly, the nonmatch would appear at the reference location in approximately half the trials (8/17 conditions). The reference location varied from session to session. ***C***, Neurophysiological recordings were performed in two cortical areas: the dlPFC, including areas 8 and 46, and the PPC, including areas 7a and LIP. IPS, intraparietal sulcus; STS, superior temporal sulcus; AS, arcuate sulcus; PS, principal sulcus.

Extracellular neurophysiology recordings were obtained from areas 8a and 46 of the dlPFC and areas 7a and LIP of the PPC of two adult monkeys trained to perform the MSNG task (Fig. 1). A total of 404 dlPFC neurons and 654 PPC neurons had sufficient trials in all MSNG conditions and were used for further analysis. The task requires the subjects to observe and remember the locations of two stimuli presented on a screen, separated by a delay period of 3 s. If the two stimuli were displayed at the same location, they defined a match trial, and the monkey should hold fixation (stay) to get a reward. If the two locations differed, then the trial was a nonmatch, and the monkey was required to make a saccade to the location of the second stimulus, which remained visible at the screen at that point (go). Possible cue locations included a reference location and eight locations deviating from the reference location by an angular distance of 11.25°, 22.5°, 45°, and 90°, clockwise and counterclockwise. The cue could be followed by a matching stimulus appearing at the same location in approximately half the trials (9/17 conditions) or by a nonmatch stimulus, which could only appear at the reference location (8/17 conditions). The reference location changed from session to session. In the MSNG task, the monkey’s response is categorical, and the saccade is always directed to the same location, thus allowing us to dissociate activity representing the location of the visual stimuli from that of motor preparation.

### PFC shows more NMS than PPC

Individual dlPFC and PPC neurons typically encode more than one variable, sometimes exhibiting NMS, which means that their response to the combination of variables cannot be predicted by the linear summation of their responses to single variables (Rigotti et al., 2013; Parthasarathy et al., 2017; Johnston et al., 2020). In the context of the MSNG task, NMS is defined as the neurons’ spatial tuning depending on whether the stimulus used to evaluate was presented in the first epoch of the task (cue) or in the second (match). This information is essential for successfully completing the task, since subjects need to know whether they should maintain stimuli into working memory or compare it with the existing contents of working memory. This action could be implemented in the form of NMS. Our analysis does not consider nonmatch stimuli, which always appeared at the same reference location, and their spatial selectivity could not be evaluated in this dataset.

**Figure 2.**
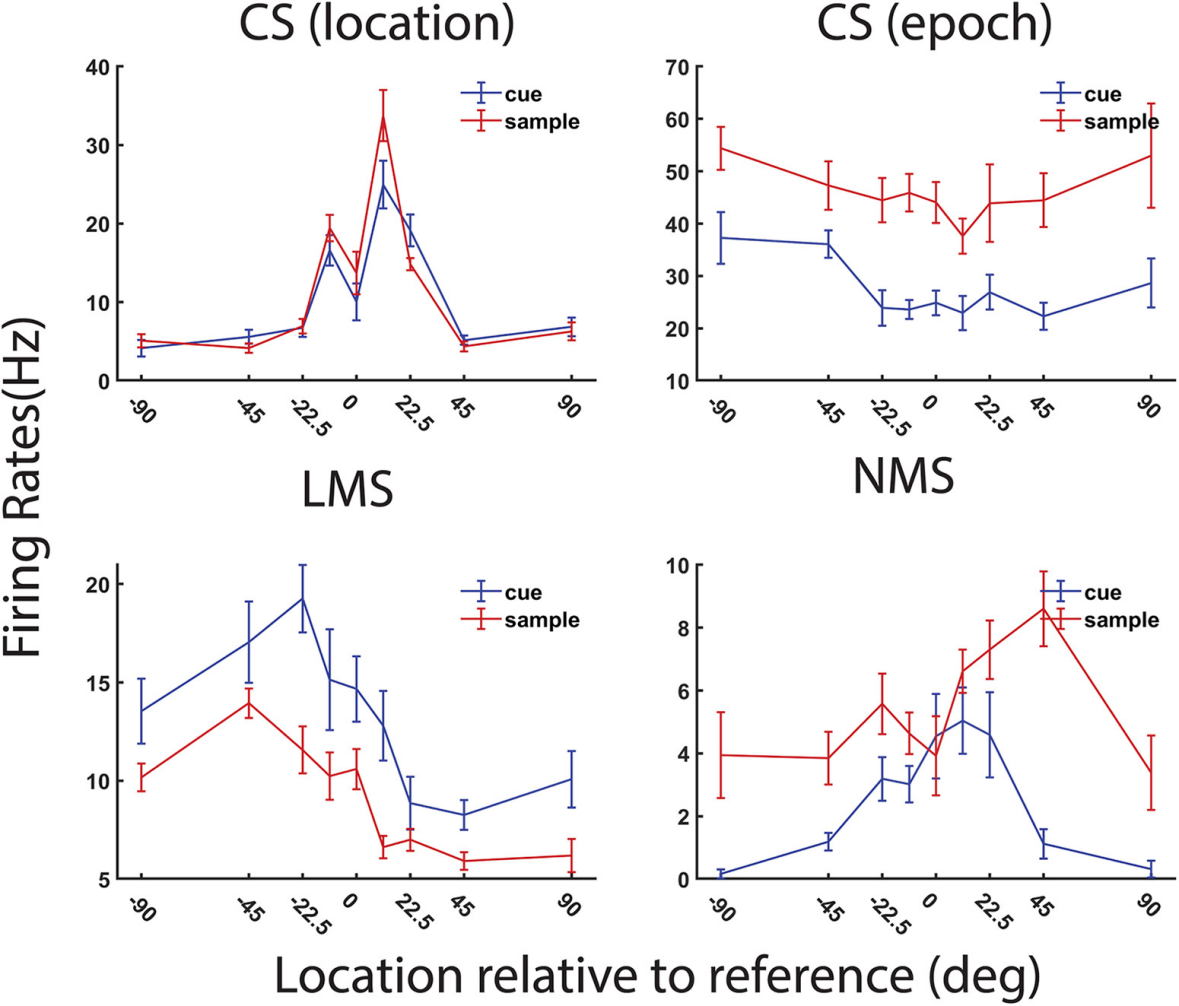
Exemplar neural responses from the MSNG task. Examples of four dlPFC neurons are shown, exhibiting CS, LMS, and NMS cells, defined by the task variables of stimulus location and task epoch. The *x*-axes represent the relative location to the center reference location. The *y*-axes represent the spiking rates during two 500-ms stimulus presentation epochs. Error bars indicate SE.

We used a two-way ANOVA with factors of stimulus location and epoch sequence to classify neurons into different categories of selectivity. CS neurons exhibited a significant main effect on only one of the factors (stimulus location or task epoch) and had no significant interaction term. In Figure 2, the first exemplar plot depicts such a CS cell, selective for location, regardless of whether the stimulus appeared as the cue or the match. The second exemplar of Figure 2 displays another CS cell not selective for the location of the stimuli but demonstrating higher mean response when the stimulus appeared as the match. Linear mixed selective (LMS) neurons exhibited a significant main effect for both factors but had no significant interaction term. The third exemplar of Figure 2 displays such an LMS neuron showing a higher mean firing rate when stimuli appear as cue while simultaneously displaying the same rank order preference for location. NMS neurons exhibited a significant interaction effect, as shown in the last exemplar in Figure 2, a neuron exhibiting different selectivity patterns for locations when shown as cue versus match.

**Figure 3.**
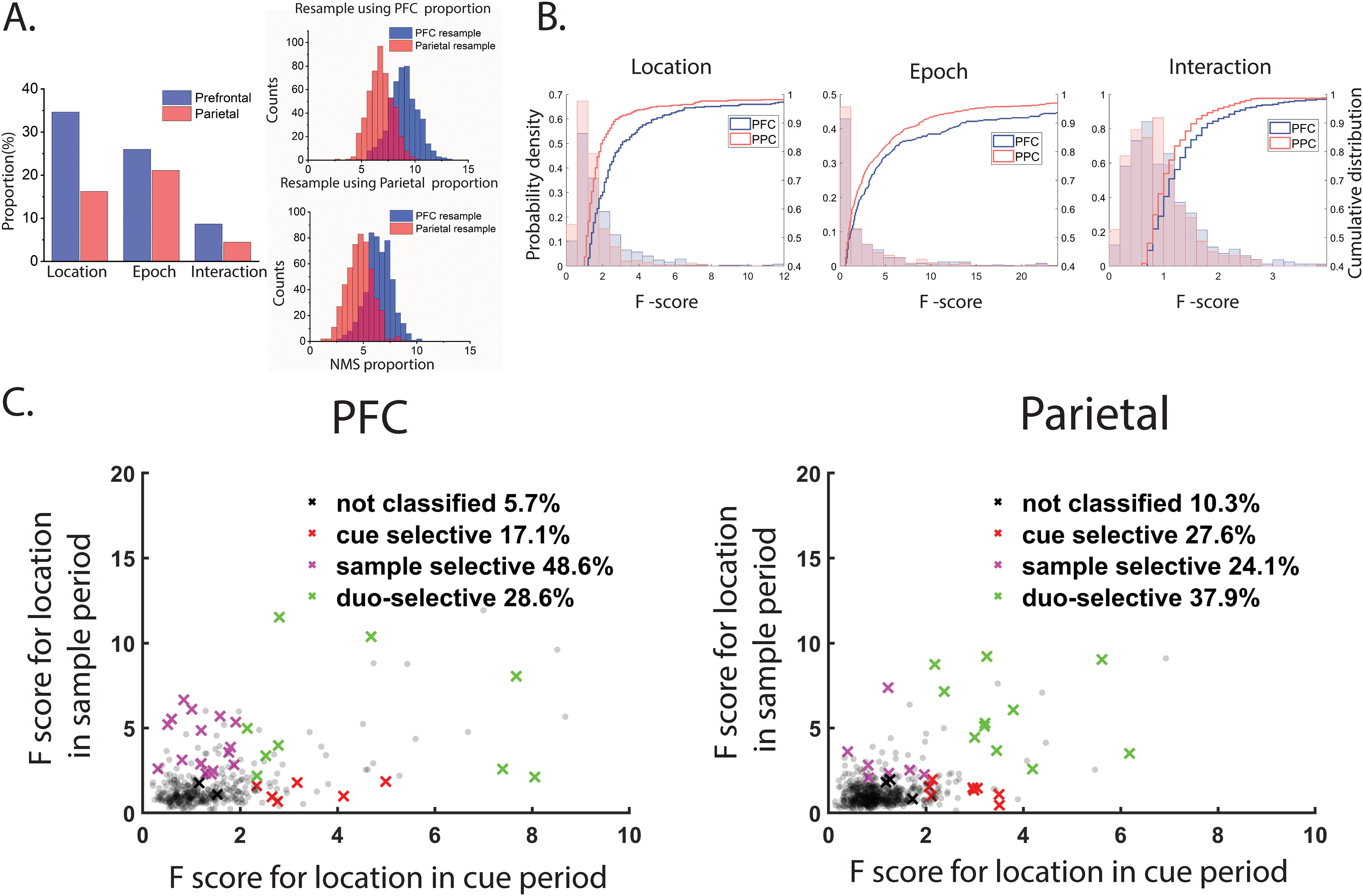
Proportion of NMS neurons in PFC and PPC. ***A***, Bar graphs show the proportions of cells tuned to stimulus location, task epoch and their interaction (i.e., NMS), in the dlPFC (*N* = 404) and PPC (*N* = 654). Increased selectivity to single task variables does not fully explain the increased levels of NMS in the dlPFC. This area continued to display a higher proportion of NMS cells compared with the PPC, even after a permutation test was used to control for different levels of CS (see Materials and Methods). ***B***, Probability and cumulative density function for the F-scores in the ANOVA test on factor of location, epoch and their interaction. ***C***, Classification of NMS cells shows that in dlPFC, the NMS is mainly contributed by cells that shows location selectivity only during the match period. All crosses (black, red, purple, and green) represent NMS cells with color coding for different selectivity categories. Gray dots indicate all other cells.

Previous research has suggested that NMS may be important for implementing complex tasks. Thus, the first question we wanted to investigate is how ubiquitous NMS is in brain areas that are involved in high order cognitive task performance. The ANOVA analysis showed that more dlPFC neurons (8.7%, 35/404) than PPC ones (4.4%, 29/654) exhibited a significant interaction term (i.e., possessed NMS) to task variables (Fig. 3*A*). dlPFC neurons in our sample were more selective overall to the stimulus, so to ensure that the higher percentage of NMS was not driven by higher overall selectivity in dlPFC, we repeated in our analysis (Fig. 3*A*), in samples of neurons equated for task-variable selectivity in the two areas. The results confirmed a higher proportion of NMS cells in dlPFC than PPC: the mean NMS cell proportion was 8.6% in dlPFC, whereas the mean NMS cell proportion was 6.6% in PPC, in a sample selected to match the dlPFC selectivity. The NMS cell proportion in the two areas was significantly different (permutation test *p* < 0.01). Resampling neurons using the PPC proportion of selectivity yielded 6.2% of dlPFC neurons and 4.5% of PPC neurons with NMS, which also constituted a significant difference (permutation test, *p* < 0.01).

We quantified and compared NMS in the two areas in an alternative manner by computing the F ratio for the interaction term of the ANOVA analysis. The mean F-ratio, computed across all neurons (not only those reached significance and were defined as NMS) was higher in the dlPFC (1.04) than the PPC (0.88). The difference between the two areas was highly significant (two-sample *t* test, *t*_(1405)_ = 3.2, *p* = 3.9 × 10^−5^).

The type of NMS we examined in our MSNG task is epoch-dependent location selectivity, and a few different profiles of responses could fall under this definition. The NMS could arise because of a shift in location preference across epochs, or because of cells that only show location selection in one of the two stimulus presentation epochs. With this intuition, we further categorized the NMS cells based on their location selection in two epochs (Fig. 3*C*). This analysis revealed that in dlPFC, location selectivity was much greater in the second stimulus period (dlPFC 17/35 = 48.6%, PPC 7/29 = 24.1%) compared with the first stimulus period (dlPFC 6/35 = 17.1%, PPC 8/29 = 27.6%). In other words, dlPFC neurons showed greater stimulus selectivity when it appeared as a match than when it appeared as a cue in the context of the task, resulting in nonlinearity in responses.

**Figure 4.**
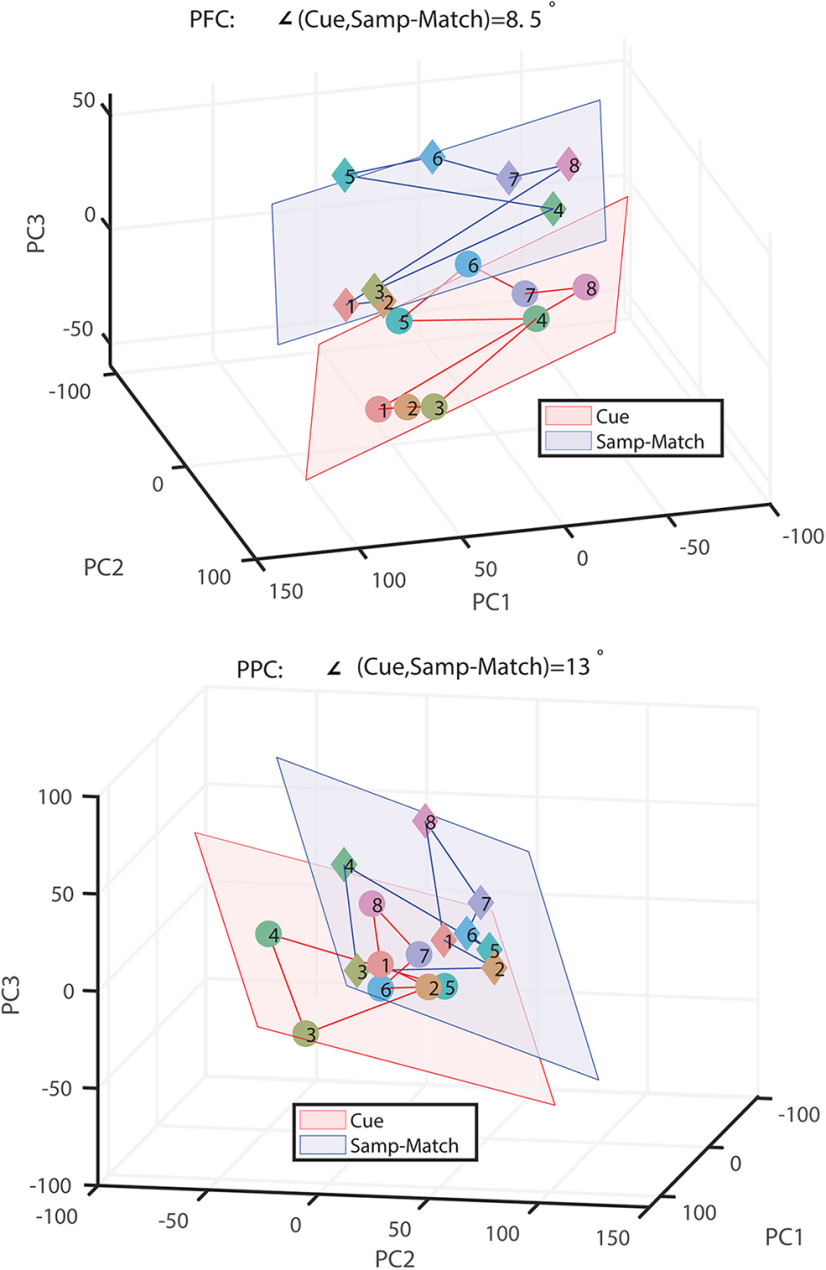
Population responses in PCA space. Each point represents responses of dlPFC (top) and PPC (bottom) neurons to one stimulus location. Numbers in points denote the eight possible locations of cue and match stimuli in Figure 1*B*, right, ordered in clockwise fashion. Red planes, Best-fit planes for the representations mean firing rate of neurons in the cue period. Blue planes, Best-fit planes in the match period. PC1, PC2, and PC3 are the first three principal components of the response space.

So far, we have analyzed NMS on the single-cell level. Previous research on dlPFC has shown that the neural representation of stimuli across the entire population exhibits transformations and dynamics (Minxha et al., 2020). We thus hypothesized that if we visualize the population response in PCA space, the transformation between the two planes defining neuronal responses in the cue and match period would be more pronounced for dlPFC. This was not the case. In both brain areas, representations of stimuli in the cue and match epochs were separable; however, in both cases, the planes that defined population responses exhibited minimal rotation (Fig. 4). The mean (and standard deviation) of the rotation angle between the cue-defined and match-defined planes was 16.9 ± 15.7° for the dlPFC and 11.3 ± 7.0° for the PPC. The results suggest that although a greater proportion of individual neurons in dlPFC alter their selectivity between epochs, the overall representation of the stimuli remained stable within a subspace in the match period.

### Changes in NMS during a single session

Although the monkeys were trained to perform the task with any combination of cue, match, and nonmatch locations, the reference location around which match and nonmatch stimuli were presented was kept the same in each recording session. Theoretically, knowing the reference location is not necessary for the task if working memory could maintain information with perfect precision. However, previous experimental studies have shown that working memory is imprecise and subject to drift over the delay period (Wimmer et al., 2014; Barbosa et al., 2020). Therefore, knowing the position of the reference stimulus could aid performance in the task, and would be expected to alter the neural representation of information in the brain. For example, under a Bayesian inference framework, to infer the posterior (match or nonmatch), one needs to accumulate data about the likelihood of the alternatives, which are expected to be reflected in the distribution of neural response for match and nonmatch conditions (Wei and Stocker, 2015).

**Figure 5.**
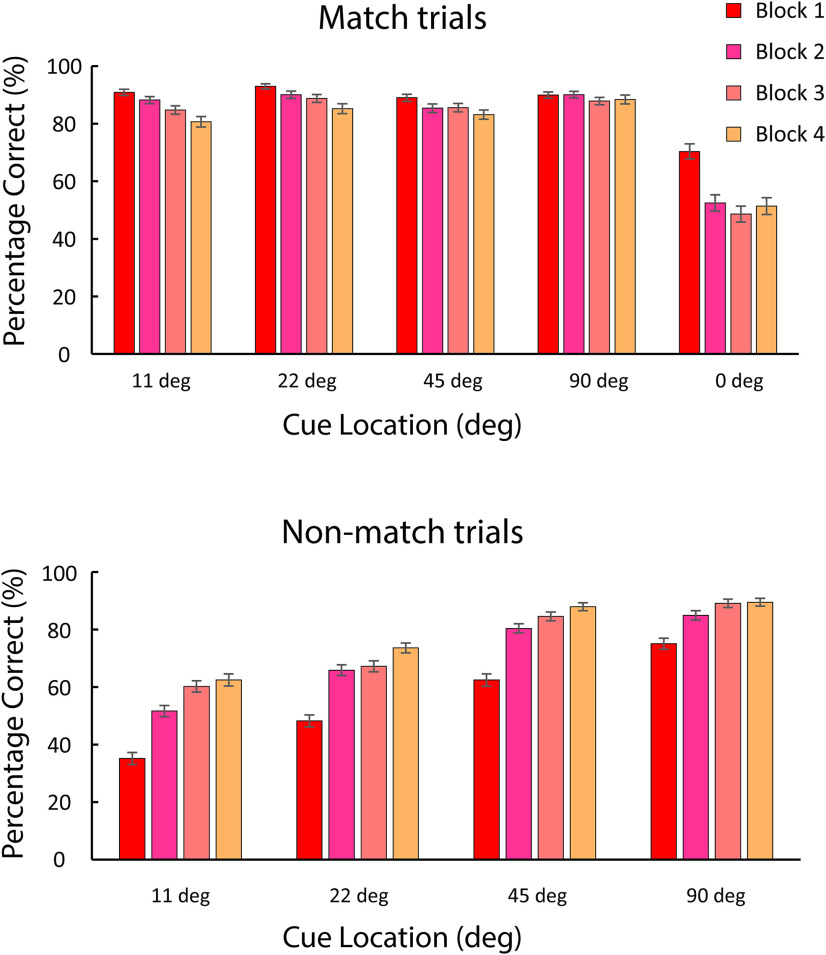
Behavior performance during each session. Mean correct behavior performance is plotted in successive 40-trial blocks. Top, Performance in match trials, when the match appeared to a position deviating from the reference location by the angle indicated. Bottom, Performance in nonmatch trials, when the cue appeared at the reference location and nonmatch stimulus appeared at the position deviating by the angle indicated. Error bars indicate SEM.

We divided single sessions into blocks of 40 trials and plotted performance in sequential blocks. As expected, the monkeys’ performance increased overall as a function of time in single sessions (Fig. 5). However, this net benefit in performance was the result of two opposing trends: performance improved for nonmatch trials deviating by small distances from the cue location, as the monkey was presumably able to recognize the nonmatch stimulus when it appeared at the increasingly familiar reference location. At the same time, performance for match stimuli appearing at the reference location declined (0° location in Fig. 5), presumably again because of the monkey’s understanding that the likelihood of any stimulus at this location being a nonmatch was low (1/9 conditions).

**Figure 6.**
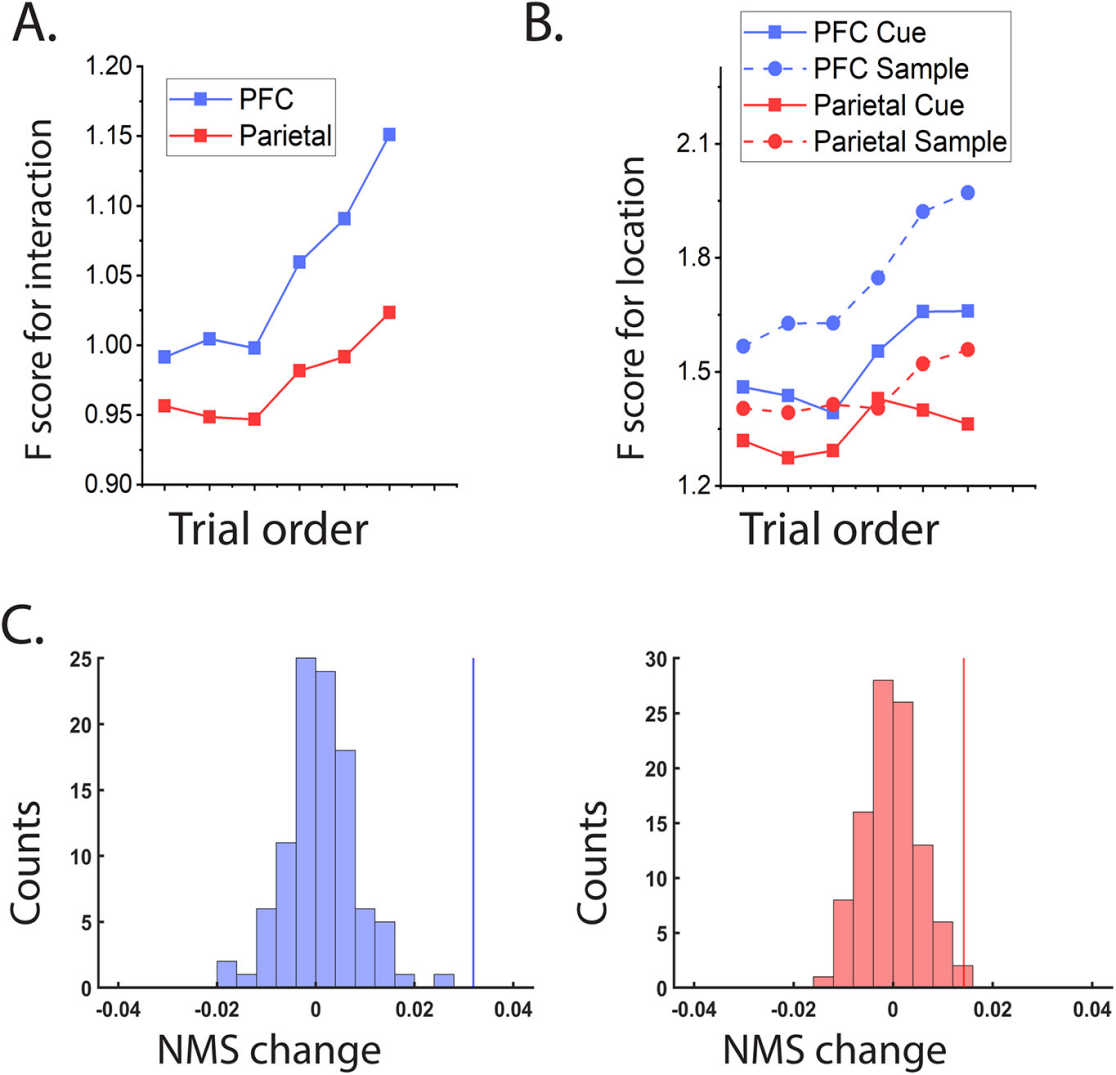
Changes in cell selectivity across a single experimental session. ***A***, Average F-score for the interaction term in the ANOVA test for dlPFC and PPC neurons plotted as a function of trial order across sessions. ***B***, Average F-score for location factor in the AVOVA in either the cue or the match period, is plotted against trial order in a session. dlPFC cells developed selectivity to location over the course of a single session more obviously, compared with PPC. ***C***, Permutation tests demonstrate that both dlPFC and PPC, displayed statistically significant changes in degree of nonlinearity, measured by F-ratio for the interaction term in ANOVA test. *x*-axes of the plots represent slopes for linear regression of trial-order-dependent F-score change. Vertical lines indicate empirical values form unshuffled data.

Since NMS has been proposed to be essential for flexible behavior in complex tasks, we wondered whether NMS changed within a single session as well. We thus analyzed neural data into blocks of trials and performed an ANOVA analysis for each block. We found that NMS significantly increased within a single session in both dlPFC and PPC (Fig. 6*A*,*C*), with a greater change in dlPFC. The mean F-score of the interaction term for dlPFC neurons was 0.99 in the first group of three trials, whereas it increased to 1.15 in the last group of three trials. In PPC, the mean F-score of the interaction term in first three trials was 0.96 and increased to 1.02 in last three trials analyzed. Further, the increase of NMS in the dlPFC was accounted for mainly by location selectivity emergence in the match period, rather than selectivity disappearance in the cue period (Fig. 6*B*). The slope of the linear regression on trial-order-dependent location selectivity was highest for the match period in the dlPFC; it was lower for the cue period relative to the match, and for the PPC relative to the dlPFC (slope values: 0.086 for the match period in dlPFC; 0.052 for the cue period in dlPFC; 0.033 for the match period in PPC; 0.021 for the cue period in PPC). In other words, neurons became more selective for the location of the match stimulus, as the reference location became more obvious, and subjects presumably relied more on the location of the match to perform the task.

### NMS in correct and error trials

**Figure 7.**
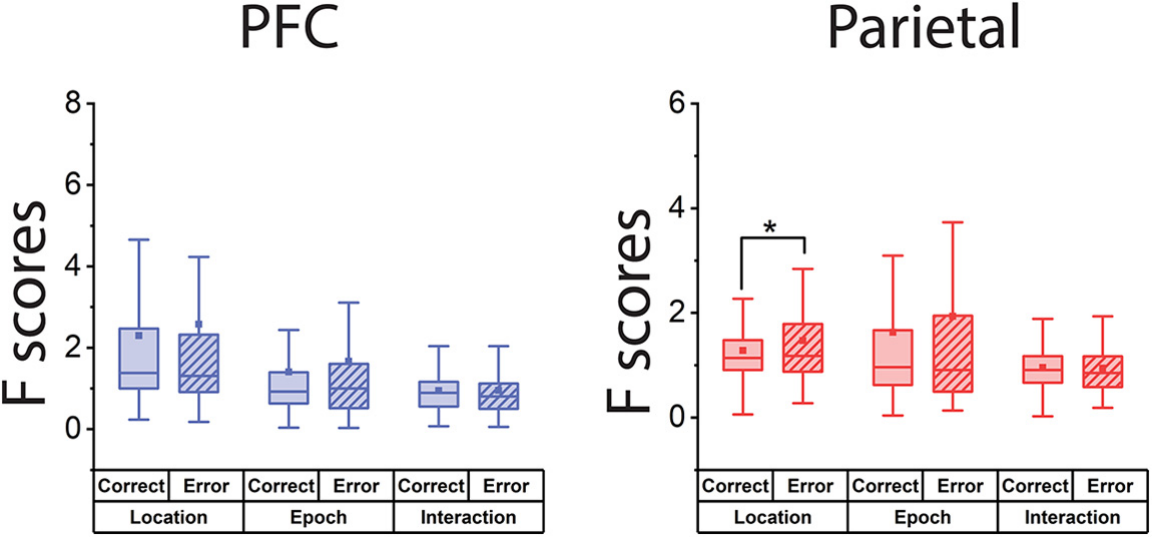
Neuronal selectivity in correct and error trials. Selectivity is plotted in the dlPFC and PPC, after controlling for the number of trials and the location pairs that were examined. No decrease in the mean F-score of the interaction term (i.e., NMS) was observed. Box boundaries represent 25−75% data range, whiskers indicate 1.5 interquartile range, and squares indicate means across each brain area.

The correlation between behavior and NMS cell proportion in dlPFC suggested that NMS may be important for successfully completing the task. If that were true, one would expect a higher level of NMS in correct trials compare to error trials. We therefore examined the F-score of the main effects and their interaction in the ANOVA test in correct versus error trials for the MSNG task (Fig. 7). The number of trials and task variables was matched for each cell to avoid confounds in the comparison. Contrary to this expectation, we did not find any decrease in the F-score of the interaction term of the ANOVA analysis. In dlPFC, the mean interaction term for correct trials was 0.95 and for error trials 0.96, a nonsignificant difference (paired *t* test, *t*_(166)_ = 0.14, *p* = 0.89), lastly. In PPC, the means for correct and error trials were 0.96 and 0.94, which also did not represent a significant difference (paired *t* test, *t*_(155)_ = 0.25, *p* = 0.81).

We actually observed a slight increase in spatial selectivity during error trials compared with controls. In dlPFC, the mean F-score in correct trials for the location variable was 2.32, while for the error trials, the mean was 2.60 (paired *t* test, *t*_(166)_ = 1.04, *p* = 0.30). In PPC, the mean F-score in correct trials for the location variable was 1.28, and 1.47 for error trials (paired *t* test, *t*_(155)_ = 2.01, *p* = 0.04). The mean F-score in correct trials for the epoch variable was 1.63, while that for the error trials was 1.93 (paired *t* test, *t*_(155)_ = 1.95, *p* = 0.05).

Similarly, there was also a modest increase in error trials for the main effect of epoch (tendency for responses during the cue epoch to be higher overall than the match epoch, or vice versa). In dlPFC, the mean F-score in correct trials for the epoch variable was 1.41 and in error trials the mean was 1.67 (paired *t* test, *t*_(166)_ = 1.90, *p* = 0.06). In PPC, the mean F-score was 1.63 in correct trials and 1.93 in the error trials (paired *t* test, *t*_(155)_ = 1.95, *p* = 0.05).

### Other forms of NMS

**Figure 8.**
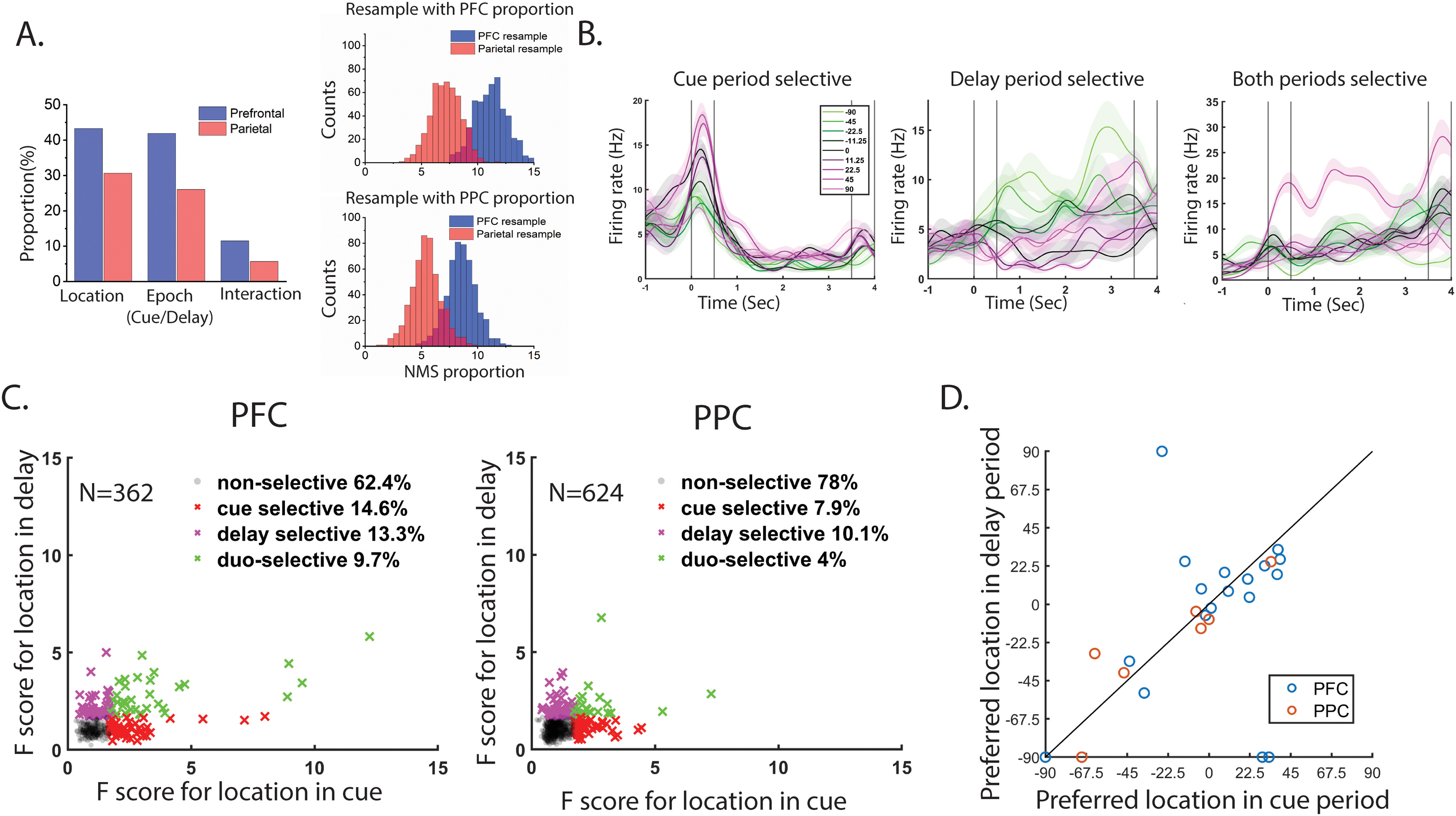
Location selectivity in the cue and the delay period. ***A***, Left, Bar graphs show the proportions of cells tuned to stimulus location, task epoch (cue vs delay) and their interaction in the dlPFC (*N* = 362) and PPC (*N* = 624). dlPFC exhibited a higher proportion of cells showing NMS (the interaction bar) defined by location selectivity during the cue and the delay period. Right, Increased selectivity to single task variables does not fully explain the increased levels of NMS in the dlPFC. This area continued to display a higher proportion of NMS cells compared with the PPC, even after a permutation test was used to control for different levels of CS (see Materials and Methods). ***B***, Three representative cells that showed cue, delay, and both period selectivity for the locations of stimuli. Shaded area represents standard error of the mean firing rate. ***C***, Scatter plot for location selectivity in the cue and the delay period, for PFC and PPC populations. ***D***, Scatter plot indicating the preferred location for the cue (abscissa) and delay period (ordinate) for cells that showed location selectivity in both periods. Each circle represents one neuron. Only neurons with responses that could be fitted by a Gaussian function were included in this analysis (*N* = 23 in PFC, *N* = 7 in PPC).

The mixed selectivity of a specific neuron could be defined in different ways, depending on what task variables were under examination. Both PFC and PPC show location selectivity in a spatial working memory task, during the stimuli presentation and the delay period (Li et al., 2021). We thus considered whether such selectivity may constitute another form of NMS, and we wanted to know how many cells maintained spatial selectivity in different task epochs. Using a two-way ANOVA as we did for the cue versus match comparison, we found that PFC had a higher degree of NMS compared with PPC (Fig. 8*A*, PFC 11.5%, PPC 5.7%). We also confirmed that the higher percentage of NMS could not be accounted for by an overall higher location selectivity in dlPFC, by equating for task-variable selectivity in the two areas in a permutation test (Fig. 8*A*). The results, again, confirmed a higher proportion of NMS cells in dlPFC than PPC: the mean NMS cell proportion was 11.5% in dlPFC, whereas the mean NMS cell proportion was 7.4% in PPC, in a sample selected to match the dlPFC selectivity. The NMS cell proportion in the two areas was significantly different (permutation test *p* < 0.01). Resampling neurons using the PPC proportion of selectivity yielded 8.9% of dlPFC neurons and 5.6% of PPC neurons with NMS, which also constituted a significant difference (permutation test, *p* < 0.01).

We additionally identified cue-period selective, delay-period selective, and both-period selective cells (Fig. 8*B*). We found that PFC had higher proportion (PFC cue-selective 14.6% vs PPC cue-selective 7.9; PFC delay-selective 13.3% vs PPC 10.1%) of cells that lose or gain spatial selectivity in different task epochs than PPC (Fig. 8*C*). For cells that displayed spatial selectivity in both the cue and the delay period, the preferred locations were comparatively stable in both areas (Fig. 8*D*), and the few neurons with large location preference changes were all found in PFC.

**Figure 9.**
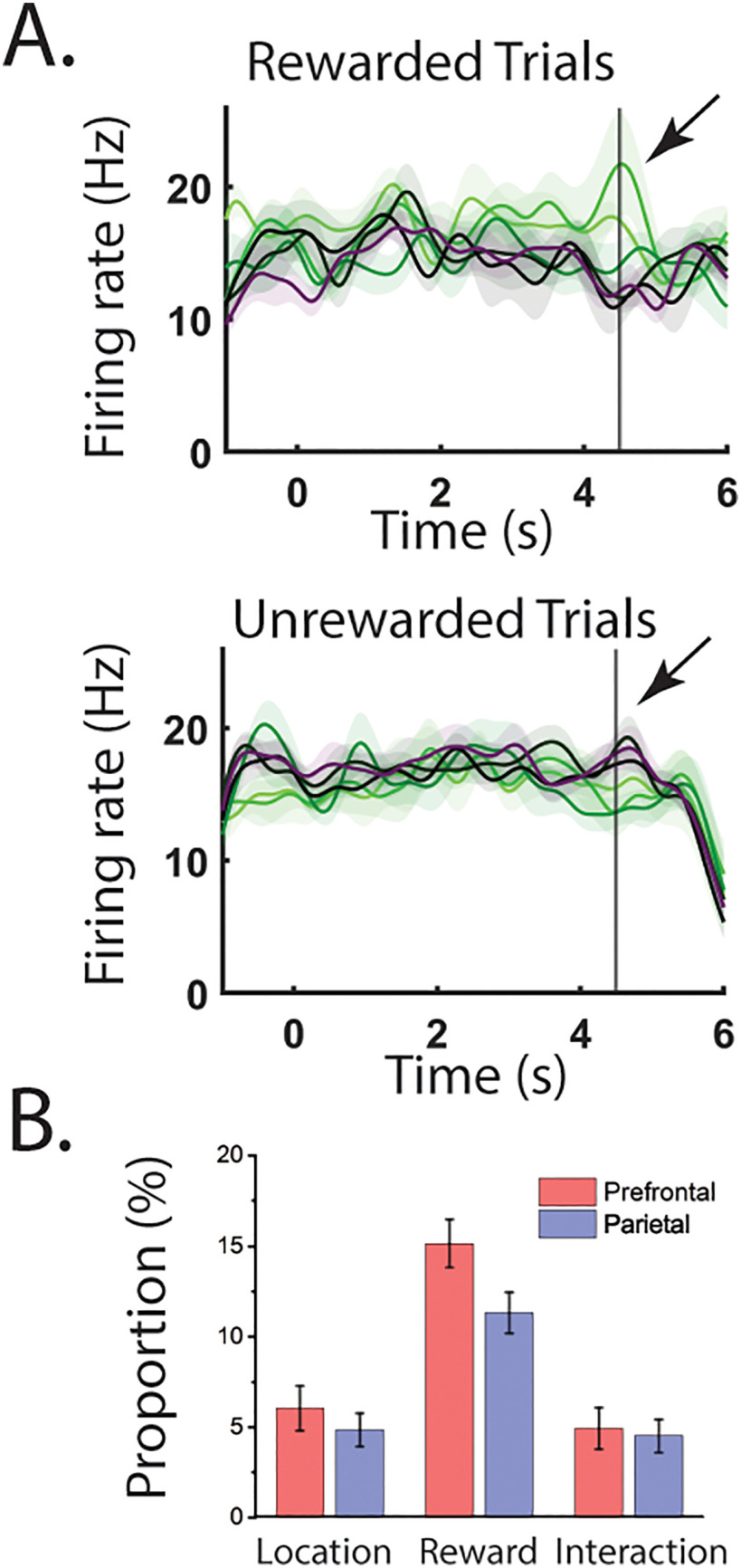
Mixed selectivity for cue location and reward. ***A***, An example cell that reversed its location preference in rewarded versus nonrewarded conditions. Black line represents the time that reward was expected. Shaded area represents standard error of the mean firing rate. ***B***, Bar graphs show the proportions of cells tuned to stimulus location, reward, and their interaction (i.e., NMS), in the dlPFC (*N* = 314) and PPC (*N* = 500). Error bar represents standard deviation in 50 resamples.

We also wondered whether the more prominent NMS we found so far in PFC was specific to pure sensory information encoding. For this purpose, we checked neural response at the end of the trial, once feedback was provided on whether the trial was correct, and reward delivered, or not. For this analysis we considered whether stimulus tuning differed depending on the correct or error status of the trial. Individual examples of cells coding both stimulus location and reward status nonlinearly at the end of the trial period are shown in Figure 9*A*. However, these cells were rare in both areas. The overall incidence of NMS for reward and stimulus location was near the chance level expected by the false positive rate of our ANOVA test, in both areas (Fig. 9*B*).

## Discussion

Our study revealed that NMS is more prevalent among dorsolateral prefrontal than posterior parietal neurons in the MSNG task, in which activity from both areas has been shown to influence spatial working memory behavior (Li et al., 2021). NMS was greater in the PFC, even after controlling for the higher overall selectivity for stimulus variables that was present in our prefrontal sample. However, even prefrontal NMS was present in only a small percentage of neurons, did not result in a substantial rotation of the population responses in PCA space, and lower magnitude on a trial-to-trial basis was not predictive of errors in task execution. These findings suggest that the role of NMS is somewhat limited and that this is not the only mechanism through which complex tasks are represented in neural activity, in agreement with other recent studies (Dang et al., 2021).

### Role of NMS

Neurons in the PFC and other areas exhibit NMS for different variables, which cannot be predicted by the linear summation of their responses to single variables (Rigotti et al., 2013; Parthasarathy et al., 2017; Johnston et al., 2020). Theoretical studies have shown that NMS is useful for linear readouts of flexible, arbitrary combinations of variables (Buonomano and Maass, 2009; Rigotti et al., 2010; Fusi et al., 2016), allowing flexible control between discrimination and generalization (Barak et al., 2013; Johnston et al., 2020). The nonlinearity can naturally emerge in recurrent networks with Hebbian plasticity (Lindsay et al., 2017). Both PFC and parietal cortex exhibit networks of recurrent connections in which the maintenance of working memory is thought to persist (Riley and Constantinidis, 2016), providing a substrate for such nonlinear selectivity to emerge.

Performance in cognitive tasks that require working memory can be improved with training (Klingberg et al., 2002, 2005; Bherer et al., 2008; Jaeggi et al., 2008; Dux et al., 2009). This malleability of cognitive performance in turn is mediated by the underlying plasticity in neural responses (Constantinidis and Klingberg, 2016; Li et al., 2020). In the context of our task, plasticity was evident in two timescales: in representing the context of a stimulus when it appeared as a cue and as a match stimulus, over the duration of a single trial; and as a result of learning the location of the reference stimulus, during the course of a daily behavioral session. NMS was evident in both of these timescales, and more so in the PFC than in the PPC. The emergence of NMS was mainly contributed by appearance of selectivity during the match period. Recent theoretical studies posit that efficient coding constrains stimulus representations to match the statistical distribution of external sensory stimuli (Wei and Stocker, 2015; Taylor and Bays, 2018; Walker et al., 2020). The behavioral and neuronal response changes we observed may reflect the process in which the dlPFC was accumulating evidence for likelihood distribution of the visual stimuli. Whatever the cause behind the learning process, NMS in dlPFC seems to correlate with this behavioral flexibility. This agrees with previously proposed role of NMS in flexible behavior.

Furthermore, we investigated NMS defined by both sensory stimuli and internal goals, to test the hypothesis that the two different areas may display NMS encoding different aspects of the tasks. In all the variable combinations we tested, we found either higher or similar levels of NMS in dlPFC compared with PPC. This may suggest that the differences in the NMS we observed are more attributed to some basic properties of the circuits, like biophysical or anatomic parameters of single cells. Other than the prevalence of NMS, it is also worthy to point out that our results revealed that some task factors seemed to be less frequently nonlinearly combined than other task factors. For example, in our task, although we found significant reward status tuning in both areas, NMS defined by reward and stimuli location was near chance level. It has been hypothesized (Rigotti et al., 2013) that NMS is beneficial for arbitrary binary classification of task conditions, thus facilitating highly flexible downstream readout and ultimately highly flexible behavior. Factors like reward status belong to the downstream readout step that may benefit from upstream NMS, since typically subjects need to make flexible decisions based on sensory inputs and strategies in mind, but the goal of behavior is rigid, namely, to obtain rewards. The subject needs a simple binary classification in terms of reward regardless of the way reward is obtained. This may explain why we observe some task factors were less involved in NMS.

Our results are consistent with another recent study from our laboratory which documented emergence of NMS in the PFC after training to perform working memory tasks for the first time, in a different cohort of animals (Dang et al., 2021). In both of these studies, the percentage of neurons that exhibited NMS was modest, and nearly absent in some task variants (Dang et al., 2021). Furthermore, lower NMS was not predictive of error trials, as would be expected by the suggested role of NMS in regulating flexible behavior (Rigotti et al., 2013). This suggests that the loss of information encoded in a nonlinear manner may not be the primary factor in failing the spatial working memory in our task. A caveat for this conclusion is that the low overall incidence of NMS may have prevented a further decline from becoming apparent, and a smaller number of error than correct trials were available for this analysis. Our population analysis also suggested that the small incidence of NMS neurons did not result in a substantial rotation of the stimulus geometry. Other studies, too, have failed to detect an appreciable percentage of neurons that exhibit NMS (Cavanagh et al., 2018).

### Prefrontal and posterior parietal specializations

The PPC and dlPFC are part of a broader network that processes visuospatial information and spatial working memory (Jaffe and Constantinidis, 2021). Tested with the ODR task, virtually identical proportions of neurons exhibiting working memory responses are found in posterior parietal and dorsolateral prefrontal areas (Chafee and Goldman-Rakic, 1998). Testing the two areas with more complex tasks, however, reveals unique properties. For example, the PFC had a greater ability to generate persistent activity representing a stimulus and suppress the response to distractors, independent of the actual temporal order of the distractor in a sequence of stimuli (Qi et al., 2010, 2015; Suzuki and Gottlieb, 2013; Jacob and Nieder, 2014). Persistent activity in the PFC is generally more robust (Masse et al., 2017) and exhibits lower levels of variability than the PPC (Qi and Constantinidis, 2012, 2015), when recordings from the same animals executing the same tasks are compared. Several studies have thus identified distinct patterns of responses and corresponding roles played by dlPFC and PPC in working memory and other cognitive functions such as categorization (Swaminathan and Freedman, 2012; Crowe et al., 2013), attentional selection (Ibos et al., 2013; Meyers et al., 2018), and response inhibition (Zhou et al., 2016). These differences in functional properties, in turn, can be attributed to differences in intrinsic properties of neurons and circuits in the two areas (Zhou et al., 2012; Katsuki et al., 2014; Hart and Huk, 2020). Most importantly, prefrontal pyramidal neurons exhibit more extensive dendritic trees and the highest numbers of spines of any cortical neurons (Elston, 2000), thus providing a substrate for greater plasticity within larger ensembles.

Our current results provide another manifestation of the greater capacity for plasticity of prefrontal neurons. It is possible that NMS appears for variables strongly represented in neural activity of an area, and in this respect the PFC may constitute a better substrate for NMS emergence in the context of this task than the PPC. Our findings are consistent with the conclusions of a recent study suggesting greater nonlinear integration in the prefrontal than the PPC, using a motion-direction task for which parietal cortex exhibited strong selectivity (Zhou et al., 2021). However, nonlinear properties were not quantified in the same manner and that study did not provide an indication of how prevalent NMS is at the level of single neurons. Our study suggested that a higher percentage of NMS neurons was evident in the dlPFC than the PPC in animals that had been trained to perform the MGNS task. Additionally, a more significant increase of NMS was evident in the dlPFC during the course of a single session. Other recent studies have also suggested stronger nonlinear integration of inputs in the PFC compared with the parietal cortex (Zhou et al., 2021). These results suggest a unique role for dlPFC in subserving working memory and flexible behavior more generally.

## Synthesis

Reviewing Editor: Mark Laubach, American University Decisions are customarily a result of the Reviewing Editor and the peer reviewers coming together and discussing their recommendations until a consensus is reached. When revisions are invited, a fact-based synthesis statement explaining their decision and outlining what is needed to prepare a revision will be listed below. The following reviewer(s) agreed to reveal their identity: Bruno Averbeck, Rei Akaishi.

Two expert reviewers reviewed your study. Their reviews are given below. Please address all points that were raised. Thank you for submitting your study to eNeuro!

Reviewer 1

In the present study, the authors compared non-linear mixed selectivity in the dlPFC and PPC. Specifically, they recorded single neurons from areas 8a and 46 of the dlPFC and 7a and LIP of the PPC while monkeys performed a Match-Stay Nonmatch-Go (MSNG) task. They found NMS neurons were more frequent in dlPFC than in PPC. The difference of NMS between these areas was mostly driven by stimulus selectivity in the match period. Interestingly, this phenome was also spotted in a single session, both in single-neuron activity and behavior.

This study investigated a question that has been discussed for a long time from a new perspective, which will be of interest to a wide range of readers interested in cognitive neuroscience. Given the unique nature of the data and its overall consistency with previous reports, I judge that the results are interpretable. However, I have some points that the authors should clarify.

Major comments:

1. To measure non-linear mixed selectivity, two variables, stimulus location and epoch have been used in the analysis. My main concern is what is the “epoch” really relevant to. Is it about time, or is it about the match/nonmatch rule? If it is all about time, why not also include the delay period since the spatial information is also maintained in working memory? Another possibility is the rule of match/nonmatch, although the authors only used match trials in their analyses due to the design of the task.

2. Related to the meaning of epoch, why do dlPFC neurons show a greater stimulus selectivity in the match period? A clearer explanation is needed.

3. What drives the change of mixed selectivity in a single session? The authors mentioned two types of learning, learning the context of stimulus presentation and learning the location of reference stimuli. I found the behavior (figure 5) somehow matches the explanation: performance in match trials indicates the learning of reference location, performance in nonmatch trials indicates the learning of context. And the learning of context, which is the discrimination of stimuli between epochs, seems slower than the other. It might be helpful to interpret the results.

Minor comments:

1. Figure 4. In the figure legend, do you mean 8 possible locations in Fig 1B-right?

2. The authors introduced the ODR task but didn’t explain what’s the task for in the study. Did you use it to select the reference location based on the recorded neuron’s receptive field? If not, you may need to mention how the reference location for each session was selected.

3. “than PPC” appeared twice in line 5 of page 13.

4. Why not group the bars in Figure 5 by deviation degree, since you want to compare the performance as a function of time (i.e., blocks), but not as a function of deviation degree.

Reviewer 2

The authors of this paper reported the latest observations related to the concept of mixed selectivity coding, in the dorsolateral prefrontal cortex and lateral intraparietal cortex. Given the importance of the concept of the mixed selectivity, its empirical examination is crucial. Since the initial report by Rigotti and his colleagues, the relevance and significance of the neurons showing this type of selectivity are mixed. Some studies report but others do not report them. Furthermore, the behavioral relevance and computational relevance of mixed selectivity is inconclusive with the not-straightforward relationship to the behavior such as correct-error analysis. The current study can be placed in this line of mixed results of the empirical test of the concept of mixed selectivity. In this study, the authors tested the regional differences of the distributions of the neurons showing mixed selectivity across the frontal and parietal areas. There have been a number of reports showing that the goal related aspects of the neuron selectivity is stronger in the dorsolateral prefrontal cortex compared to the lateral intraparietal cortex. The question is whether the same thing is true for the mixed selectivity analysis.

Major points:

1. The observation that the NMS is more prevalent in the dorsolateral prefrontal cortex may be dependent on the specific variables included in the analysis. For example, more goal directed aspects of the variables can be more prominently represented in the dlPFC. Before making the sweeping conclusion that the NMS is more prevalent in dlPFC, is there any way to test the generality of this claim? The lateral parietal areas are more sensitive and spatial aspects of the task variables. Is it possible to include the variables that can be more easily coded in the lateral parietal regions?

2. Related to the first point, dlPFC would be more tuned to the goal-directed aspects of the task structure. In fact, the latter epoch more closely related to the goal (reward delivery) are more strongly encoded in the data. Is it possible to classify the variables into goal-directed and non-goal-directed types and then to analyze the data with regard to NMS?

Minor points:

1. The introductions in the manuscript uses the examples of multiple tasks for the existence of the neurons with mixed selectivity. The current task does not use such multiple task context and does not directly test this idea. Some other ways of introduction might be more appropriate.

2. The focus of the paper is more on the regional difference between the lateral frontal and lateral parietal areas. The authors might need to give more examples of the previous studies showing the differences in functions and physiological characteristics of these brain areas.

3. It seems that there is accumulating evidence that the mixed selectivity is ubiquitous and/or straightforwardly related to behaviors. The authors might wish to add some discussions of why certain variables are coded more easily in mixed selectivity and other variables are not coded so easily. This would give some clue about the principles of information coding in these higher-order brain areas.
